# A durable murine model of spleen transplantation with arterial and venous anastomoses

**DOI:** 10.1038/s41598-020-60983-7

**Published:** 2020-03-04

**Authors:** Jose-Luiz Figueiredo, Fernando Santa-Cruz, José Luiz Lima-Filho, Ingo Hilgendorf, Masanori Aikawa, Mikael J. Pittet, Matthias Nahrendorf, Ralph Weissleder, Filip K. Swirski, Clinton S. Robbins

**Affiliations:** 1 Department of Surgery, Experimental Surgery Unit, Federal University of Pernambuco, Recife, Pernambuco Brazil; 2 Medical School, Federal University of Pernambuco, Recife, Pernambuco Brazil; 3 Laboratório de Imunopatologia Keizo Asami (LIKA), Federal University of Pernambuco, Recife, Pernambuco Brazil; 4 Department of Cardiology and Angiology I, Heart Center, University of Freiburg, Freiburg, Germany; 5 Cardiovascular Division, Brigham and Women’s Hospital, Harvard University, Boston, Massachusetts USA; 6 Center for Systems Biology, Massachusetts General Hospital and Harvard Medical School, Boston, Massachusetts USA; 7 Department of Systems Biology, Harvard Medical School, Boston, Massachusetts USA; 8 Department of Radiology, Massachusetts General Hospital, Boston, Massachusetts USA; 9 Departments of Laboratory Medicine and Pathobiology and Immunology, University of Toronto, Toronto, Ontario Canada; 10 Toronto General Research Institute, University Health Network, Toronto, ON Canada; 11 Peter Munk Cardiac Centre, Toronto, ON Canada

**Keywords:** Organ transplantation, Experimental models of disease

## Abstract

The spleen is a large lymphoid organ located in the abdomen that filters blood and regulates the immune system. The extent of mobilization of splenic immune cells to peripheral tissues in health and disease, however, remains poorly understood. This is due, in large part, to a lack of *in vivo*, spleen-specific lineage tagging strategies. Here, we describe a detailed practical protocol of spleen transplantation and its evaluation for long-term graft survival. Unlike implantation of splenic morsels in the great omentum, our approach uses arterial and venous anastomoses which rapidly restores blood flow and facilitates long-term survival of the graft. The use of congenic mouse strains permits the use of immunofluorescence and flow cytometry-based methodologies to unambiguously track the migration of spleen-derived cells to peripheral tissues.

## Introduction

Located within the abdomen of most vertebrates, the spleen is a large lymphoid organ known for its function in filtering the blood (ie. recycling iron and removing damaged and senescent red blood cells) and regulating the immune system^1–3^. Numerous immune cells reside within the organ, different subsets of T and B lymphocytes and myeloid cells among them^4^. Due to lack of *in vivo* cell labeling strategies, the extent of mobilization and function of splenic immune cells in peripheral tissues remains poorly understood. The most common loss-of-function approach is surgical removal of the organ, a procedure that indiscriminately eliminates all splenic cells. Splenectomy has additional effects including increased circulating white blood cell and platelet counts and heightened susceptibility to bacterial and protozoal infections^5–7^. To circumvent these issues, we developed a murine model of spleen transplantation with arterial and venous anastomosis. Surgical transplantation between congenic mouse strains (e.g. animals that differ at the CD45.1 and CD45.2 alleles) not only preserved tissue integrity but allowed unambiguous and quantitative tracking of the movement of immune cells between the spleen and peripheral tissues.

## Materials and Methods

### Ethics approval

This study was approved by the Institutional Animal Care and Use Committee (IACUC) at the Massachusetts General Hospital under the protocol number 2009N000022. The protocol as conforms to the USDA Animal Welfare Act, PHS Policy on Humane Care and Use of Laboratory Animals, the “ILAR Guide for the Care and Use of Laboratory Animals” and other applicable laws and regulations.

### Spleen transplantation protocol

#### Preparation and removal of donor spleen

**Figure 1:**
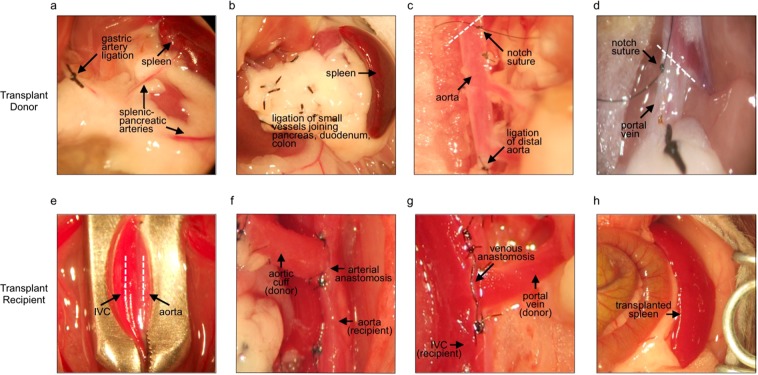
Spleen transplantation protocol. (**a–d**) Preparation of the donor spleen for transplantation. Visualization of the splenic-pancreatic arteries and ligation of the gastric artery (**a**). Ligation of the small vessels joining the pancreas, duodenum, and colon (**b**). Visualization of the donor aorta showing position of double ligature of the distal aorta and nylon notch suture of the external angle at the tip of where the proximal aorta is cut (dotted white line) (**c**). Visualization of donor portal vein showing notch suture placed at external angle of tip of where portal vein is cut (dotted white line) (**d**). (**e–h**) Spleen transplantation recipient. Placement of microvascular clamp on the aorta and inferior vena cava of transplant recipient (**e**). Visualization of completed arterial anastomosis between donor aortic cuff and recipient aorta (**f**). Visualization of completed anastomosis between donor portal vein and recipient inferior vena cava (**g**). Image showing transplanted donor spleen 1 month after the procedure (**h**). The spleen appears healthy and well perfused.

Anesthetize the donor mouse (CD45.1^+^ C57BL6/J) with an intraperitoneal (i.p.) injection of ketamine (90 mg/kg)/xylazine (10 mg/kg) using a 30-G needle. Once the donor mouse is anesthetized (this usually takes about 5 minutes), secure the animal on an acrylic movable surgical table using surgical tape. Shave and disinfect the anterior abdominal and thoracic walls using a combination of iodine and alcohol prep pads. To access the heart for whole body perfusion, make a transversal incision on the left thoracic wall at the 4^th^ intercostal space with surgical scissors. Retract the ribs, open the pericardium, and cut a small hole in the right atrium. Using a 23-G needle and 5 ml syringe, slowly inject 5 ml of cold saline solution containing 300 units of heparin into the left ventricle. CRITICAL STEP: When perfusing, do not flush all blood. Leave behind a small volume of heparinized blood for better visualization of donor vasculature. To visualize the donor spleen, pancreas, and abdominal vasculature in the epigastric region, make a longitudinal ‘V’ incision on the anterior abdominal wall extending from the pelvis to the inferior ribs. CRITICAL STEP: To prevent donor tissue from drying out, place gauze soaked in cold saline solution over the intestines, pancreas and spleen. Keep gauze in place until the spleen isolation is completed. Using 6–0 silk suture, ligate the gastric artery as well as the small vessels between the pancreas, duodenum and colon **(**Fig. 1a,b**)**. CRITICAL STEP: Be sure to handle the pancreas gently, since pancreatic trauma negatively affects transplantation outcome. Using 10–0 nylon suture, ligate the hepatic and superior mesenteric artery. Make double ligatures of the distal aorta directly below the mesenteric artery and above the right renal artery using 10–0 nylon suture **(**Fig. 1c**)**. Ligate the 4 small posterior spinal-aortic arteries with 10–0 nylon suture. Cut the proximal aorta below the diaphragm at an internal angle of 45 degrees toward the right kidney, placing a 10–0 nylon notch suture on the external angle at the tip of the open aorta **(**Fig. 1c**)**. Cut the distal aorta below the double ligatures, above the right renal artery. Isolate and ligate the common bile duct and the pancreatic-biliary vessels using 10–0 nylon suture. Cut the portal vein below the first bifurcation at an internal angle of 45 degrees towards the right hepatic lobe and place a 10–0 nylon notch suture on the external angle at the tip of the open portal vein **(**Fig. 1d**)**. Using cotton tip applicators, gently remove the organ package containing the spleen, pancreas, and vascular connections from the abdominal cavity and place it in a small petri dish containing ice cold saline while the recipient mouse is being prepared.

#### Transplantation of donor spleen into recipient

Anesthetize the transplant recipient mouse (CD45.2^+^ C57BL6/J) with isoflurane supplemented with oxygen (2–3 Vol%). Once anesthetized, remove the animal from the induction box and secure to a small, movable cork surgical table by the tail with surgical tape. Do NOT tape arms and legs. Maintain anesthesia during surgery using an inhalation apparatus. Administer preemptive analgesia via subcutaneous injection (5 mg/kg Meloxicam). Shave the anterior abdominal wall and disinfect the skin using a combination of iodine and alcohol prep pads. Cover the operating field with a sterile fenestrated surgical drape. Make a mid-line incision on the abdominal wall and open the peritoneal cavity. Retract both sides of the abdominal wall with a sterilized modified paper clip. Pin the clips down on the cork table with bent 23-G needles. To visualize the recipient’s spleen, gently mobilize the intestines to the right side of the anterior abdominal wall using cotton tip applicators and cover them with saline solution soaked gauze. Remove the orthotopic CD45.2^+^ spleen by ligating the splenic vessels with 6–0 silk suture and excising the organ. Cover the liver, stomach, and pancreas with gauze soaked with ice cold saline solution. Cut the avascular tissue that connects the descendent colon to the pancreas with fine micro-scissors and gently release the infra-renal aorta and inferior vena cava (IVC) from surrounding tissues using fine forceps and cotton tips. CRITICAL STEP: Be careful not to damage the sciatic nerves that run parallel to the aorta and IVC. Place a microvascular clamp on the aorta-IVC below the renal vessels **(**Fig. 1e**)**. CRITICAL STEP: Be sure not to clamp sciatic nerves. CRITICAL STEP: To have enough space to perform anastomosis, make sure at least 10 mm of vessel is pinched within the clamp. Using a 30 G needle, puncture the proximal area of the isolated aorta. Using this opening as a starting point, make a 2 mm incision of the clamped aorta using fine micro-scissors. Flush out any micro-clots with ice cold saline **(**Fig. 1e**)**. Position the donor spleen and pancreas on the left side of the abdomen and make an anchorage notch on the inferior angle of recipient aorta using 10–0 nylon suture. Using 10–0 nylon suture, begin a running terminal-lateral anastomosis (joining donor and recipient aortas) with one stop from the superior angle of opened right side of aorta. Gently move the donor spleen and pancreas to the right side of the abdomen using cotton applicator tips and continue the running anastomosis on the left side of aorta **(**Fig. 1f**)**. CRITICAL STEP: Do not over-tighten the running aortic anastomosis. Using cotton applicator tips, move the spleen carefully to the left side of the abdomen. Then, using a 30-G needle, puncture the distal end of clamped IVC. Using this opening as a starting point, make a 2 mm incision of the clamped IVC using fine micro-scissors **(**Fig. 1e**)**. Flush out any micro-clots with ice cold saline. Make an anchorage notch on the inferior angle of recipient opened IVC with 10–0 nylon suture. Start a running terminal-lateral anastomosis (joining the donor portal vein with the recipient’s IVC) with one stop from the superior angle of the opened right side of the IVC. Move the spleen carefully to the right side of the abdomen and continue the running anastomosis on the left side of IVC **(**Fig. 1g**)**. CRITICAL STEP: Do not over-tighten the running vein anastomosis. Once the anastomoses are completed, open the vascular clamp slowly to perfuse the spleen. If minor bleeding in the splenic and/or pancreatic vessels occurs, ligate leaky vessels with 10–0 nylon suture. Once certain all bleeding has stopped, gently place intestines back inside the peritoneal cavity and close the abdominal wall using absorbable 6–0 vicryl suture. Place the recipient mouse in a clean cage, under a heat lamp until the animal regains consciousness.

TIMING: Preparation and removal of donor spleen and transplantation of donor spleen into the recipient should be kept to 40–50 minutes each.

NOTE: A detailed list of all required reagents and equipment can be found in Supplementary Materials.

### Ultrasonagraphy

Short and long term blood perfusion of transplanted spleens was confirmed by high resolution ultrasonography using Vevo 2100 system (FujiFilm VisualSonics, Toronto, ON, Canada).CD45.2^+^ C57BL6/J transplanted recipient mice were anesthetized with isoflurane supplemented with oxygen (2–3 Vol%). Once anesthetized, animals were removed from the induction box and placed in dorsal position on a heated imaging platform and remained anesthetized via a nose cone. Prior to start imaging, the tail vein was catheterized, using a 27-gauge needle connected to a modified P10 tubing catheter, and the anterior abdominal wall was shaved. Aquasonic-100 ultrasound transmission gel (Parker Laboratories, Fairfield, NJ) was applied over the abdominal wall and a 40-MHz imaging transducer was placed longitudinally over the left region of the abdomen. B-mode and color doppler imaging were taken before and after a bolus tail vein injection of 100-μL of microbubbles contrast agent (VEVO non-targeted micromarker microbubbles, FujiFilm VisualSonics) and continue microbubbles injection at a constant rate of 9 μL/min through the duration of spleen imaging.

### Flow cytometry

Single cell preparations were stained for 30 minutes on ice unless otherwise noted before being fixed with Cytofix buffer (BD Biosciences). CD45^+^ hematopoietic spleen cells were identified as: CD11b^+^IAb^+^CD11c^+^ (dendritic cells; DCs), CD11b^+^IAb^+^CD11c^−^Ly6C^+^/^−^ (monocytes), Lineage^−^CD11b^−^F4/80^+^CD115^low^ (red pulp macrophages), CD11b^+^IAb^−^CD11c^−^Ly6C^intermediate^ (neutrophils), CD19^+^IgM^low^CD23^+^ (B2/Follicular B cells), CD19^+^IgM^high^CD23^−^ (Marginal zone/B1 B cells), CD3^+^CD4^+^ (CD4 T cells), CD3^+^CD8^+^ (CD8 T cells), and CD3^−^NK1.1^+^ (NK1.1^+^ cells). Data were acquired on a LSR II (BD Biosciences) and analyzed using FlowJo software (v.10; Tree Star). Positive and negative gating strategies were determined using isotype controls unless otherwise stated.

### Histology

To assess splenic architecture, spleens were resected and fixed in 4% paraformaldehyde, cryoprotected in 20% sucrose in PBS before being embedded in Tissue-Tek optimal cutting temperature compound (Sakura Finetek), and flash frozen in isopentane on dry ice. 5-μm transverse sections were made and stained with hematoxylin and and eosin (H&E).

## Results

**Figure 2:**
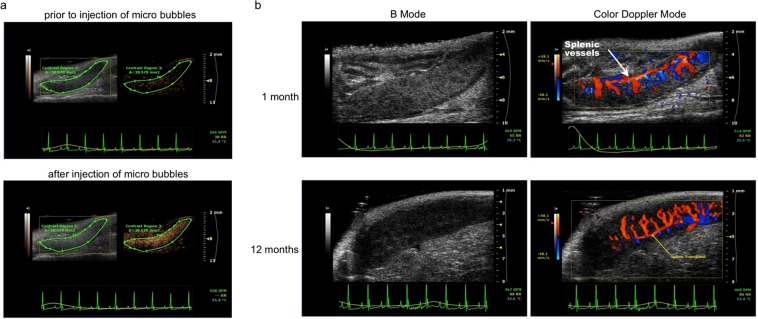
Spleen transplantation with venous and arterial anastomoses results in long term survival of the transplanted organ. Visualization of spleen perfusion with contrast ultrasound 1 week after transplantation (**a**). Left panels show B-mode scans. Right panels show representative contrast scans. Top 2 panels show transplanted spleen prior to injection with microbubble contrast agent. Bottom 2 panels show transplanted spleen after injection with contrast microbubbles. Colored doppler ultrasound scans showing blood flow in the spleen 1 month (top panels) and 12 months (bottom panels) following transplantation (**b**). Oxygenated arterial blood is red. Deoxygenated venous blood is blue.

**Figure 3:**
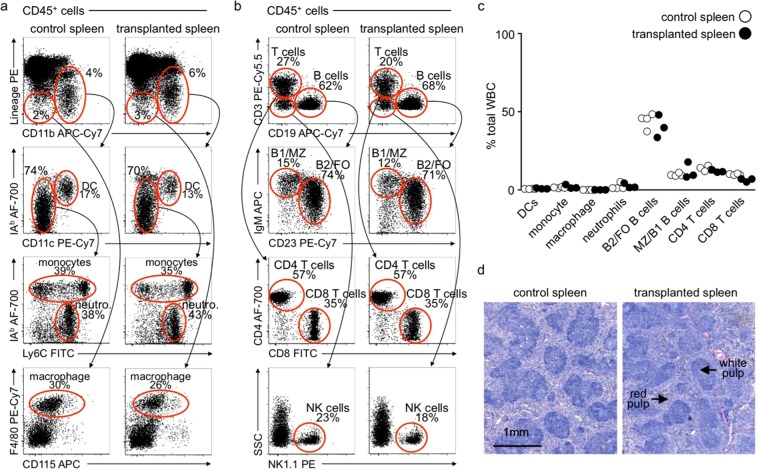
Splenic immune cell composition one year following transplantation. Spleens from 12 week old donor C57BL6/J mice were transplanted into 12 week old recipient C57BL6/J mice. One year later spleens were harvested from transplant recipients as well as age matched non-transplanted control mice. (**a**) Flow cytometry dot plots depict gating strategies to identify several myeloid cell populations including splenic dendritic cells (DC), monocytes, neutrophils, and macrophages. Transplantation had no effect on the relative percentage of all myeloid cells analyzed (**a**). (**b**) Flow cytometry dot plots depict gating strategies to identify lymphoid cell populations including splenic CD4 and CD8 T cells, B1 and marginal zone (MZ) B cells, B2 and follicular (FO) B cells, and natural killer (NK) cells. Transplantation had no effect on the relative percentage of all lymphoid cells analyzed (**b**). (**c**) Data depicts percentages of immune cells in (**a**) and (**b**) as a percentage total white blood cells. Individual animals are shown. *n* = 4 (control), 3 (spleen transplant). (**d**) H&E staining reveals normal red and white pulp architecture in transplanted spleens one year after the procedure.

Spleens from 12 week old donor C57BL6/J mice were transplanted into 12 week old recipient C57/BL6/J mice (see detailed protocol in Materials and Methods, Fig. 1) and several parameters were used to assess overall fitness of the transplanted organ over 1 year. First, transplanted spleens appeared healthy. Immediately after and at several time points to 1 year following transplantation, spleens presented as characteristically purplish red suggesting the organ was adequately perfused **(data not shown and** Fig. 1h**)**. In previous studies we demonstrated perfusion of the transplanted spleen using a combination of Angiosense-680 injection, a fluorescent blood pool agent, and near infrared imaging (NIR)^8^. Here, we confirm long term blood perfusion of transplanted spleens by contrast ultrasonography (Visual Sonics Vevo^®^ 2100 System) at 1 week (Fig. 2a) and colored doppler ultrasound at 1 and 12 months following transplantation (Fig. 2b). We next determined whether our transplant approach affected the physiological distribution of immune cells within the spleen. Flow cytometry studies revealed no difference in the proportion of myeloid cell subsets including dendritic cells, monocytes, and macrophages between transplant recipients and non-transplanted controls 1 year following transplantation **(**Fig. 3a,c**)**. Similarly, transplantation had little effect on the distribution of several subsets of B and T lymphocytes and natural killer (NK) cells **(**Fig. 3b,c**)**. Histological analysis of transplanted spleens revealed normal architecture of the red and white pulp structures **(**Fig. 3d**)**. Collectively, the data show that our approach preserves tissue integrity and is associated with long term survival of the transplanted organ.

**Figure 4:**
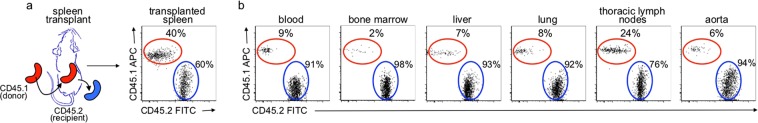
Steady state trafficking of splenic leukocytes between congenic mice following spleen transplantation. Spleens from 12 week old donor CD45.1 C57BL6/J mice were transplanted into 12 week old recipient CD45.2 C57BL6/J mice. Two days later the transplanted spleen as well as several peripheral tissues (blood, bone marrow, liver, lung, thoracic lymph nodes, aorta) were harvested and single cell suspensions analyzed by flow cytometry to distinguish spleen donor (CD45.1^+^)− and transplant recipient (CD45.2^+^)−derived leukocytes. Spleen−derived leukocytes were observed in all tissues analyzed. One representative experiment is shown.

To demonstrate how our approach can be used to track the migration of splenic immune cells, spleens from 12 week old donor CD45.1 C57BL6/J mice were transplanted into 12 week old recipient CD45.2 C57BL6/J mice. Two days later, the proportion of donor (CD45.1^+^) and recipient (CD45.2^+^) cells was assessed in several organs of the transplant recipient including the blood, bone marrow, liver, lung, thoracic lymph nodes and aorta. Our data demonstrate that steady state trafficking of immune cells in and out of the spleen is remarkably dynamic **(**Fig. 4a**)**. Moreover, CD45.1 spleen-derived leukocytes migrated to all of the peripheral tissues analyzed **(**Fig. 4b**)**. How splenic leukocyte migration is altered in the context of different pathological conditions remains to be determined.

## Discussion

### Overview of the technique

The approach involves splenic transplantation between congenic (CD45.1 and CD45.2) mouse strains which are identical but for one molecule expressed on the surface of white blood cells that can be identified by antibody detection. Therefore, immunofluorescence and flow cytometry methodologies can be used to distinguish hematopoietic cells derived from the CD45.1 donor spleen and CD45.2 recipient. A key aspect of our approach is the creation of arterial and venous anastomoses between donor and host, resulting in rapid restoration of blood flow and long-term survival of the spleen graft. Vascular anastomosis to the recipients’ circulation is facilitated by transplanting the donor splenic artery and vein with a section of the abdominal aorta and portal vein, respectively. The CD45.1 donor organ package, which includes the spleen, a portion of the pancreas, and vascular connections is then transplanted into the peritoneal cavity of the CD45.2 recipient using end-to-side anastomoses of the donor aortic cuff to the recipient descending aorta, and of the donor portal vein to the recipient inferior vena cava. To achieve spleen exchange rather than supplementation, the endogenous CD45.2 spleen is removed.

### Applications and target audience

The approach has broad applications—it can be used to determine the fate and function of various cell types migrating in and out of the spleen. The use of congenic mouse strains allows clear identification of donor and host cells in any tissue of interest. The method is therefore of interest to all scientists studying inflammation and immunity in health and disease. In 2009, Swirski *et al*. demonstrated that the spleen is a monocyte reservoir^9^. In this study, our spleen transplantation protocol revealed splenic monocytes mobilize in response to myocardial infarction, accumulate in the injured heart, and participate in wound healing. Using the approach, we have also showed that the spleen is an active site of extramedullary hematopoiesis during atherogenesis^8^. We determined that monocytes produced in the spleen contribute to disease progression by infiltrating atherosclerotic lesions and differentiating to foamy macrophages, secreting inflammatory cytokines, reactive oxygen species and proteolytic enzymes. In a genetic mouse model of lung adenocarcinoma, Cortez-Retamozo and colleagues used the transplantation protocol to establish that neutrophils and macrophages borne in a splenic niche relocate to the site of the tumor and promote cancer growth^10^. Collectively, these studies demonstrate that our spleen transplantation method is an effective *in vivo* approach for labeling spleen cells and assessing their function in a remote tissue context. Of note, our previous studies followed graft survival to ~1.5 weeks. Here, we report graft survival, perfusion, and normal distribution of immune cell subsets in the transplanted spleen out to one year post transplant, demonstrating the utility of the approach for long-term studies. The procedure can be used in different pathological settings and possible modifications include transplantation of spleens from donors in which certain genes are either knocked out or over expressed.

### Comparison with other techniques

Implantation of spleen morsels into the great omentum of mice has previously been reported^11^. However, the approach does not incorporate vascular anastomoses which leads to prolonged ischemia and eventual resorption of the transplanted tissue. Furthermore, lack of an adequate blood supply precludes the study of physiologic cell trafficking. Transplantation of the intact spleen with arterial and venous anastomosis, therefore, represents a major technical advance, not simply an extension of previous implantation strategies. A recent video publication of spleen transplantation with venous and arterial anastomosis in mice utilized cauterization of the short gastric vein attached to the spleen^12^. In many jurisdictions, the use of vessel cauterization when harvesting organs for transplant in animals is prohibited. Moreover, cauterization often leads to inflammation, injuring the vessels that are required to be ligated to the stomach, pancreas, colon and duodenum. Here, we demonstrate that vessel ligation leads to durable survival of the graft long-term.

The most commonly used approaches to study immunological processes in the spleen include splenectomy and/or adoptive transfer of single cell suspensions prepared from tissue homogenates. Splenectomy restricts controlled investigation of specific subsets of immune cells since removal of the organ at once eliminates *all* cell populations. Injection of single cell suspensions from spleen homogenates removes cells from their physiological tissue context. Our spleen transplantation protocol avoids these issues, allowing spleen cells to be unambiguously marked without disturbing the tissue environment in which they reside.

## Supplementary information

Supplementary Information.
