# Supplementary material for: A durable murine model of spleen transplantation with arterial and venous anastomoses
Source: Sci Rep. 2020 Mar 4;10:3979. doi: 10.1038/s41598-020-60983-7 (PMC7055260; doi:10.1038/s41598-020-60983-7)
Supplement: Supplementary file 1 — Supplementary Information. [file 41598_2020_60983_MOESM1_ESM.pdf]

Supplementary Information

A durable murine model of spleen transplantation with arterial and venous anastomoses

Jose-Luiz Figueiredo, Fernando Santa-Cruz, José Luiz Lima-Filho, Ingo Hilgendorf, Masanori Aikawa, Mikael J. Pittet, Matthias Nahrendorf, Ralph Weissleder, Filip K. Swirski, and Clinton S. Robbins

MATERIALS

Reagents

- Ketamine (100mg/mL; Webster Veterinary, cat. no. 07-869-6095)
- Xylazine (100mg/mL; Butler Schein, cat. no. 37849)
- Isoflurane (VWR, cat. no. 95045-588)
- Meloxicam (5mg/mL; Patterson Veterinary, cat. no. 07-890-7338)
- Heparin (1000 USP Units/mL; Sagent Pharmaceuticals, Butler Schein, Cat: 33185)
- Saline solution (0.9% NaCl; Hospira Inc. Owens and Minor, cat. no. 22-415-685)
- Mice, CD45.1 and CD45.2 congenic C57BL/6J (The Jackson Laboratory; cat. no. 0664)

!CAUTION All animal studies must be reviewed and approved by the institutional animal care and use committees and conform all relevant ethics regulations.

CRITICAL All experiments must be performed under sterile conditions. Researchers should wear sterile hats, gowns, and gloves.

Equipment

- Alcohol prep pads (Fisher Scientific; cat. no. 22-415-685)
- Povidone-Iodine Swabstick (PDI; cat. no. S41125)
- 3M Transpore Surgical Tape (Fisher Scientific; cat. no. 18-999-381)
- Sterile Gauze Sponges, 5cmx5cm Sterile (Tyco Healthcare; Ref. 1806)
- Cotton Tip Applicator (Puritan Medical Products, VWR; cat. no. 89176-684)
- Polystyrene petri dishes, 100 x 15mm (VWR; cat. no. 25384-302)
- Fenestrated Surgical Drape (VWR; cat. no. 300008-591)
- Modified Paper Clip (available from any office supply)
- 6-0 silk suture (ETHICON; cat. no. 1639G)
- 10-0 nylon suture (ETHICON; cat. no. 2870G)
- 6-0 Vicryl (ETHICON; cat. no. J499G)
- 6-0 nylon suture (ETHICON; cat. no. 697G)
- 5mL BD syringes (VWR; cat. no. BD309646)
- 1mL BD tuberculin syringe (VWR; cat. no. 309659)

- 23G Needle (Becton Dickinson; cat. no. 305143)
- 27G Needle with attached tuberculin syringes, 0.5mL (Becton Dickinson; cat. no. 305620)
- 30G Needle (Becton Dickinson; cat. no. 305128).
- 30G Needle (30G Blunt Needle 0.5in. VWR; cat. no. 89134-188)
- Microscope (SMZ645 Zoom Stereomicroscope, Nikon)
- Cold light source (KL 1500, Leica, Schott)
- Anesthesia inhalation apparatus (Forane Vaporizer Model: 100, SurgiVet)
- Induction Box (Sure-Seal Induction Chamber, Braintree Scientific; cat. no. EZ-177)
- Stopcock for isoflurane apparatus (Smiths Medical 4-Way Stopcock w/ Swivel Male Luer Lock, Fisher Scientific; cat. no. NC9052592)
- Air Filter for isoflurane apparatus (F/Air Filter 80120 - Bickford - Carbon canister, Webster Veterinary cat. no. 78010225)
- Acrylic movable surgical table (Cooks Direct, Carlisle 6" x 9" x 1/2" White Cutting Board, cat. no. CA1901 WHITE)
- Forceps (Fine Science Tools; cat. no. 91106-12)
- Surgical scissors (Fine Science Tools; cat. no. 91460-11)
- Needle holder (Fine Science Tools; cat. no. 91201-13)
- Fine forceps (Fine Science Tools; cat. no. 91150-20)
- Micro-scissors (Fine Science Tools; cat. no. 15003-08)
- Microvascular Bulldog Clamp (ASSI; cat. no. 2815326V)
- Cork table (4 x 12" cork sheet, VWR; cat. no. 23420-708)
- Surgical Mini Clippers (Roboz Surgical Instruments; cat. no. RC-5903)
- Procedure face mask (VWR; cat. no. 47080)
- Isolation gown (Fisher Scientific; cat. no. 19-160-256)
- Basic protection bouffant cap (VWR; cat. no. 89131-622)
- Heat Lamp (Braintree Scientific; cat. no. HL-1 US)
